# Surgery

**Published:** 1903-03

**Authors:** J. Lacy Firth


### SURGERY.

The immediate and remote results of **partial and complete excision of the larynx for malignant disease** have of late years been considerably better than they were formerly. Not only has the mortality of these operations been diminished, but, in addition, the degree of freedom from recurrence of the disease following them, and of perfection in the functional after-results, have been increased.

Frank Hartley, of New York,[1] has recently contributed a valuable article to the literature of this subject, and verified the foregoing statements by reference to the published statistics, both old and new. In addition, he has described some of the means by which the improved results have been gained.

In this, as in most other departments of surgery, many statistical tables have been drawn up by various authors, which are open to the objection that they present the results in too favourable a light; for while human nature remains what it is, successful cases will continue to find their way into statistical tables, and unsuccessful ones, which should be there, will find a way of keeping out, and of being consigned to oblivion.

Hartley gives, in chronological order, a summary of all the important statistics hitherto published bearing on this subject; but we cannot say that it is easy from these to gain clear ideas as to what exactly have been the relative results of the four chief operative methods used for the removal of the disease in question in its varying grades. That, however, is the kind of information the surgeon needs, in order to decide which operation should be recommended in a given case, and with what

[1] *N. York M. J.*, 1902, lxxvi. 1020, 1059.

prospects. The four operative measures we allude to are: (1) The endo-laryngeal, in which the growth is removed through the mouth by laryngological means; (2) that by thyrotomy (laryngo-fissure), *i.e.* by dividing the thyroid cartilage in front, and removing the soft parts from within it; (3) that by partial excision of the larynx, including the framework as well as the soft parts; and (4) that by complete extirpation of the larynx.

On the basis of the statistics he quotes, Hartley writes: "The diminution in the death rate for laryngectomies from 1889 to 1900 was from 44 per cent. to 8.5 per cent. or 14 per cent. The increase in those remaining cured over three years has been from 7 to 15 or 16 per cent. during this period. The increase in those remaining free from recurrences, but not over three years, has been from 13 or 14 per cent. to 33 per cent. during the same period." In the class of cases dealt with by thyrotomy, or partial removal of the soft parts, he states that to-day the permanent cures are as high as 44 per cent., while the death-rate can be placed at 11 per cent., if two cases of accidental death in von Bergmann's clinic are excluded. We shall add below some conclusions of Butlin as to the results which are to be expected from this latter mode of operating. It will be seen, therefore, that our opening statement, that of late there had been an all-round improvement in the results of operations for malignant disease of the larynx, appears to be abundantly justified by statistics.

For a comparison of the relative results obtained by each of the four methods of operating described above, we append, arranged in a tabular form, the statistics given by Sendziak[1] in 1899, and quoted by Hartley.[2] They are based upon a collection of 640 cases.

| Nature of Operation. | No. of Cases. | Mortality. | Definitely Healed. | Recurrences. | Unknown Results. |
|---|---|---|---|---|---|
| Endo-laryngeal ... ... | 36 | *Nil* | 39 % | 39 % | 22 % |
| Thyrotomy .. ... ... | 136 | 2 % | 25 % | 59 % | 16 % |
| Partial Laryngectomy. | 201 | 21.8 % | 23 % | 31.3 % | 23.9 % |
| Complete Excision ... | 267 | 35 % | 13 % | 30 % | 22 % |

We give this table because it is based upon such a large number of cases, practically all the cases that Sendziak could collect from literature. But the figures do not accurately show the present-day status of the operations under consideration, for they are materially influenced by the inclusion of the earlier cases, in which, as we have shown above, the results were so much worse than those now obtainable. In 1896 Sendziak[3] published a similar collection of cases, including only those up to the year 1894, and the statistics based on those cases have been

[1] *Jahresb. f. Chir.*, 1899, 480. [2] *Loc. cit.*, 1021.
[3] *Die bösartigen Geschwulste des Kehlkopfes*, Wiesbaden, 1897.

carefully considered by Butlin.[1] The later statistics present most of the difficulties of interpretation which Butlin encountered in dealing with the earlier statistics. For example, the early cases are not separated from the modern ones, nor the cases of intrinsic from those of extrinsic carcinoma. By English writers much importance is attached to this latter distinction; for they hold strongly the view that much better operative results follow in the cases of intrinsic carcinoma,—that is, in those which primarily affect the true or false vocal cords, the ventricles, or the parts below the cords,—than in the cases of extrinsic carcinoma; for in the former there is little tendency for the disease to implicate either the framework of the larynx or the lymphatic glands, except in the very advanced stages. For these cases, therefore, the less serious operations, such as thyrotomy or partial excision, are often the best. In the extrinsic cases, on the other hand, those in which the growth primarily affects the epiglottis, the ary-epiglottic folds, or the arytenoid region, the disease soon spreads to other regions,—to the pharynx, tongue, tonsil, or palate,—and soon attacks the lymphatic glands; and the removal of the growth, therefore, is usually a much more formidable undertaking than in the other class of cases, and the immediate and remote results are proportionately worse.

In further criticism of the methods of operation under discussion, we would first quote with approval Butlin's recommendation that though, sometimes, brilliant results have been obtained by removing the disease *per vias naturales*, yet this method is too uncertain, and should be employed only in very exceptional cases, and only in those in which the disease is very limited, and quite on the edge of the vocal cord, and in which old age and bad health preclude other operations. By this method the growth is not likely to be efficiently removed, and there is a possibility by irritation of increasing its activity. With regard to the operation of thyrotomy, Butlin[2] writes: "I feel quite certain that one hundred thyrotomies ought to be performed at the present time for intrinsic carcinoma with a mortality of three or four, perhaps even less than this." Discussing the results of the operation, the same writer states that up to the end of 1896, Sir Felix Semon and he had performed the operation on sixteen patients, and that ten of them were alive and free from the disease for at least three years after the operation, and that the results of their later operations promised to be quite as good.

The best immediate results hitherto recorded from complete excision of the larynx are those of Glück,[3] who, in 1900,

1 *The Operative Surgery of Malignant Disease*, 2nd Ed., 1900, p. 195.

2 *Op. cit.*

3 *Verandhl. d. deutsch. Gesellsch. f. Chir.*, 1900. Quoted by Hartley.

reported 34 cases with 31 recoveries—a mortality of only 8.5 per cent.

The causes which have led to the improved results now obtainable from these operations on the larynx are to be attributed to the improved technique, by which the former frequent causes of death—in particular, aspiration-pneumonia, and infection of the cellular tissne around the trachea and continuous with that in the mediastina—are more effectually guarded against. The chief measures now adopted which are beneficial in the above way are the following, all of which are alluded to by Hartley:—

(1.) The avoidance of general anæsthesia and the more frequent employment of local anæsthesia. Kocher has used cocaine more extensively than anyone, with the object of preventing cough, stilling hemorrhage, and diminishing pain. Further, by using cocaine the reflex irritability of the trachea and bronchi seen in general anæsthesia is avoided. The cocaine also leads to a better definition of the limits of the growth (Butlin), and it diminishes the tendency to sudden reflex inhibition of the heart and respiration through the superior laryngeal branch of the pneumogastric nerve (Crile). For the latter purpose also, the subcutaneous injection of $\frac{1}{100}$ gr. of atropine has been recommended.

(2). The use of the Trendelenburg and Rose posture for the patient during the operation, in which the natural tendency of all secretion and blood is to flow away from the trachea. By this posture the danger of the aspiration of these secretions is diminished. Also, in the more restricted operations, with the patient in this position, tracheotomy may be dispensed with, and in all cases the various tampon cannulæ may be avoided, the use of which is said rather to increase than prevent the danger of aspiration pneumonia.

(3.) The complete division of the trachea at the level of the upper rings in total laryngectomy, and its deflection forwards and suture to the skin in the lower part of the wound. To accomplish this it may be necessary to divide the thyroid isthmus in front, and to separate the œsophagus from the trachea behind. This plan, adopted and recommended by Glück in 1881, appears to be a very valuable one, tending to diminish the danger of infection of the lungs from the discharge of the mouth and pharynx, and to prevent infection of the cellular tissues of the neck from the tracheal discharges. Another advantage is, that the subsequent use of a tracheal cannula is sometimes avoided, for the lumen of the trachea does not undergo stenosis.

(4.) The danger of infection of the peri-tracheal connective tissue in total extirpation of the larynx can be much diminished by shutting off the cavities of the mouth and pharynx from the wound, and from the lumen of the trachea. This may be effected in various ways. Bardenheuer, in 1891, recommended the preservation, as far as possible, of the mucous membrane of the anterior wall of the œsophagus, and that of the ary-epiglottic folds, and the suturing of these surfaces together. If necessary, he made use of the inverted epiglottis, suturing its freshly-pared edges to the walls of the pharynx and the anterior wall of the œsophagus. The wound was packed with gauze and allowed to granulate. Ritter, in addition to the above, sutured the divided muscles over the mucous membrane and the skin, as far as possible over all. Sacchi endeavoured to close the lumen of the upper end of the trachea, by suturing together the mucous membrane of the anterior wall of the œsophagus, the sides of the pharynx and the skin-flaps, over the end of the trachea. Glück, in his later cases, after resection of the trachea, closed the wound by superimposed skin-flaps taken from the sides of the wound, in a manner like that used for urethral fistula. Fœderle, in one case, after extirpation of the larynx, was able to loosen the trachea and unite its cut end to the hyoid bone, and join the mucous membrane of the trachea to the epiglottis and ary-epiglottic folds. This excellent procedure has not apparently been successfully repeated. Hartley tried to accomplish it in one of his cases, but failed. In another reported case it was accomplished; but in a few days all the sutures gave way from tension.

(5.) The performance of thyrotomy as an early step in all cases in which laryngectomy is contemplated is strongly advised. By this means the growth can be thoroughly examined, and the best means of dealing with it decided upon; and in that way unnecessarily severe operations avoided.

(6.) The immediate result of all these operations is much influenced by judicious after-treatment. Hartley adopts the plan of raising the foot of the bed during the first three days, and keeps the patient without a pillow. Rectal feeding is used until the third day, and then the patient allowed to try and swallow when lying in an exaggerated Trendelenburg position, trying to swallow, as it were, "uphill." If the wound is not closed, some leakage may occur; and if this cannot be prevented by external pressure, a catheter must be used; but it is better to avoid that instrument, if possible. In this position of the patient, any escaping fluid tends to flow away from the trachea. Marmaduke Sheild,[1] in cases of thyrotomy, recommends the patient to be kept in the sitting posture in a "steam tent," and to have creosote inhalations, and a spray of Listerine and water occasionally, and not to use the voice at all for a full month. The first attempts to swallow, are made

[1] *Brit. M. J.*, 1902, I. 941.

with the patient on the side, with the tube of the feeder in the dependent cheek.

In spite of bestowing the most careful attention upon all the details described above, both during the operation and subsequently, partial and complete laryngectomy remain operations of great severity, and entail a heavy mortality. The cases which call for these procedures will usually be associated with glandular involvement. It appears to be generally held, that good results cannot be obtained, and that the serious operations should therefore be given up in those cases in which the deep cervical and peritracheal glands are implicated. Only in the cases in which the glands affected are those in the pre- and lateral-laryngeal regions can the disease be satisfactorily removed.

Lastly, to illustrate the degree of functional recovery which may possibly be attained after complete excision, we may briefly refer to three very successful cases. In 1895, at the British Medical Association Annual Meeting in London, Solis Cohen showed a patient, brought over from Philadelphia, in whom, after extirpation of the larynx, the tracheal stump had been fixed to the lower part of the skin incision. The patient could swallow with ease and comfort, and could speak through the open mouth of his trachea so distinctly that he could be heard and understood at the end of a long room. In a case of Schmidt's also, the patient acquired a voice, which was thought to be made between the base of the tongue and the wall of the pharynx. Lastly, Goldstein[1] has had a case in which, by training, the voice became so developed that the patient could be heard at a distance of three or four feet. The trachea in these cases was not in communication with the oro-pharynx. The voice was believed to be produced between the pharyngeal wall and the remains of the epiglottis. In the latter cases, the air necessary to pass through the narrow slit representing the new larynx, was believed to be first collected in a dilatation of the pharynx below the tongue or epiglottis and above the œsophagus. A second, or reserve dilatation, was thought to be in the upper fourth of the œsophagus (Hartley). Such cases as these seem to show that in the future the artificial larynx may cease to be used, a desirable thing, for such instruments act as irritants, and in some cases have been thought to encourage recurrence of the growth; and, further, they are difficult to keep clean and in order.

J. Lacy Firth.

[1] *Verhandl. d. deutsch. Gesellsch. f. Chir.*, 1900.

